# Effects of Ultrasound and Microwave–Hot Air Drying on the Drying Kinetics and Physicochemical Properties of Mango Powder

**DOI:** 10.1155/ijfo/6693603

**Published:** 2025-12-29

**Authors:** Ahmed Abdirahman Ahmed, Gökçen Yıldız

**Affiliations:** ^1^ Department of Agriculture, Faculty of Agriculture & Natural Resources, City University of Mogadishu, Mogadishu, Somalia; ^2^ Department of Food Engineering, Faculty of Engineering and Natural Sciences, Bursa Technical University, Bursa, Turkey, btu.edu.tr

**Keywords:** hot air drying, mango powder, microwave, pretreatment, ultrasound

## Abstract

This study evaluated the effects of ultrasound (ULS) pretreatment (15 and 30 min) combined with different drying methods—hot air drying (HAD), microwave drying (MW), and hybrid microwave–hot air drying (MW‐HAD)—on the drying kinetics and physicochemical attributes of mango powder. Results demonstrate that 30 min ULS pretreatment with MW‐HAD (200 W + 70^°^C) significantly enhanced drying efficiency, reducing processing time by 80% (*p* < 0.05). However, quality outcomes were method‐dependent: MW drying at 200 W without pretreatment optimally preserved bioactive compounds, maintaining phenolic content (1431.86 mg/100 g GA dry weight) and antioxidant capacity (23.41 *μ*mol Trolox/g dry weight). Conversely, HAD at 70°C without pretreatment produced powders with superior physical properties, including higher bulk density (0.35 g/cm^3^) and improved flow characteristics (CI = 33.35). Functional properties were most enhanced by ULS pretreatment, which increased water solubility index (42.32%) and swelling capacity (48.91 mL/g). These findings provide a framework for industrial process selection: MW drying for nutrient retention, HAD for powder handling, and ULS pretreatment MW‐HAD for time‐sensitive production. The study emphasizes the importance of method selection based on targeted quality parameters in mango powder processing. Furthermore, future research should focus on optimizing ULS parameters to simultaneously maximize efficiency and quality, facilitating the industrial adoption of this promising technology.

## 1. Introduction

Ultrasound (ULS) refers to mechanical waves with frequencies between 20 kHz and 100 MHz that can travel through solids, liquids, and gases [1, 2]. These waves induce vibrations that alter the physical and structural properties of materials. In the 18–500 kHz range, ULS causes the “sponge effect,” where alternating compression and expansion create microchannels in cellular structures, promoting the release of intracellular fluids. Additionally, ULS applied in liquid media induces cavitation—bubble formation and collapse driven by pressure waves—resulting in localized high‐pressure, high‐temperature conditions that cause cellular damage [3].

Mango (*Mangifera indica* L.) is a highly perishable fruit that is susceptible to spoilage because of its high moisture content. Drying is widely used to extend shelf life by reducing moisture, inhibiting microbial growth, and lowering product weight and transport costs [4]. Nevertheless, conventional drying methods are often time‐ and energy‐consuming, prompting interest in advanced techniques. Hybrid drying methods, such as combining vacuum or convective drying with electrotechnologies like MW, radio frequency, or infrared heating, offer improved efficiency [5]. Despite this potential, these advanced methods are not without limitations, including high energy use, quality loss in HAD, overheating in MW drying, and high costs in freeze and hybrid drying [6].

ULS pretreatment is widely used to reduce the initial moisture content and modify tissue structure, thereby enhancing drying efficiency. High‐intensity, low‐frequency ULS disrupts cell membranes, accelerating mass transfer between intracellular and extracellular environments [7]. For instance, [8] reported a 27.3% reduction in the drying time of jambo fruit (*Syzygium malaccense* L.) after ULS pretreatment. Similarly, [9] noted a fivefold increase in the drying rate of blackberry (*Rubus glaucus*) using ULS before HAD. Other studies have shown that ULS pretreatment can also affect bulk density and improve the retention of bioactive compounds like phenolics [10, 11].

ULS pretreatment has been extensively investigated for its applications in various food processing operations, including defoaming, freezing, extraction, emulsification, hydration, and drying [12]. However, the combined effect of ULS pretreatment with modern drying methods like microwave and hybrid systems for mango processing remains less explored. Therefore, this study examined the effects of ULS pretreatment combined with different drying methods on the drying kinetics and selected physicochemical attributes of mango powder.

## 2. Materials and Methods

### 2.1. Sample Preparation

Fresh mangoes (*Mangifera indica* L.) at a commercially ripe stage were purchased from a local market in Bursa, Turkey. The fruits were selected based on uniform peel color, similar size, and the absence of physical defects. They were transported to the laboratory and stored at 4^°^C ± 0.5^°^C until the experiments commenced. For drying, the mangoes were cut into square‐shaped pieces with an edge length of 2 ± 0.02 mm and a thickness of 1.78 ± 0.03 mm.

### 2.2. Experimental Design

The mango slices were assigned to nine drying treatments, which included applications with and without ULS pretreatment. For the ULS pretreatment, samples were immersed in an ultrasonic bath (Daihan WiseClean WUC‐D10H, Korea) operating at a frequency of 40 kHz, a power of 400 W, and an amplitude level of 75%. Pretreatment was conducted at room temperature for two durations: 15 and 30 min. The samples were placed in a beaker with distilled water at a sample‐to‐water ratio of 1:4 (w/v). Following pretreatment, the samples were drained and blotted with filter paper to remove excess surface water before drying.

Drying was performed using a hybrid microwave–hot air oven (Arçelik KMF 833, Turkey). The samples were subjected to three drying conditions: HAD at 70°C, MW at 200 W, and MW‐HAD combining 200 W with 70°C. The microwave power of 200 W was selected based on preliminary experiments, which provided superior visual and microstructural characteristics compared to other tested powers. This choice is supported by literature suggesting that power levels of 150–200 W optimally balance drying efficiency with the retention of bioactive compounds [13].

All drying experiments were conducted in duplicate. During the process, the samples were weighed at 10‐min intervals using a digital balance with a precision of 0.01 g (Denver TP‐3002, Germany). After drying, the samples were ground at a constant speed and passed through a 60‐mesh sieve to obtain a uniform mango powder.

### 2.3. Moisture Content Determination

The moisture content at any time *t* during drying of mango samples was determined using the following equation [14]:

Mt=m−DMDM

where the moisture content at any given time, *M*_t_, was defined as the ratio of the weight of water to the weight of dry matter in the sample. In this equation, *m* is the sample weight in grams, and DM denotes the dry matter content of the sample.

### 2.4. Drying Rate Analysis

The drying rate was determined based on the derivative of the drying time curve with respect to moisture content, following equation [14]:

Drying rate=Mt+dt−Mtdt

where *M*_t_ + *d*_t_ represents the moisture content at time *t* + *d*_t_ (grams of water per gram of dry matter), *M*_t_ represents the moisture content at time *t* (grams of water per gram of dry matter), and *d*_t_ denotes the drying time interval (minutes).

### 2.5. Physical and Powder Properties

The bulk density (*ρ*_bulk_) and tapped density (*ρ*_tapped_) were determined according to the method of [15]. For *ρ*_bulk_, mango powder samples were freely poured into a 50‐mL glass measuring cylinder and weighed using a precision balance. The *ρ*_tapped_ was determined by tapping the powder sample 100 times in a graduated cylinder and recording the resulting volume. The particle density (*ρ*_particle_) was measured by dispersing 1 g of powder in 5 mL of petroleum ether in a 10‐mL capped cylinder. Subsequently, an additional 1 mL of petroleum ether was added to rinse the cylinder walls. The total volume was then recorded, and the particle density (*ρ*_particle_) was calculated using a specific formula:

Particle density ρparticle=powder product weight g total volume of petroleum ether and suspended product mL−6.

The porosity, flowability, and cohesiveness of the mango powder samples were evaluated using porosity (*ε*), Carr index (CI), and Hausner ratio (HR), respectively. The equations used to calculate *ε*, CI, and HR are as follows:

ε=ρparticle−ρtappedρparticle×100,CI=ρtapped−ρbulkρtapped×100,HR=ρtapped−ρbulk.

The swelling capacity of the mango powder samples was determined according to the method of [15]. Initially, the volume (*V*_1_) of a 1 g powder sample (*M*) was measured. Subsequently, 10 mL of distilled water was added, and the resulting mixture was shaken. The wetted sample was then allowed to settle at room temperature for 48 h, after which the final volume (*V*_2_) of the swollen product was recorded. The swelling capacity (milliliters per gram) was calculated using the following equation:

Swelling capacity mL/g=V2−V1M.

The water solubility index (WSI) of the mango powder samples was determined as described by [15]. A 0.8 g sample (*S*_1_) was mixed with pure water at a ratio of 0.02:1 (w/v) and incubated at 80°C for 30 min in a Memmert WNB 14 water bath (Germany). The mixture was subsequently centrifuged at 25°C for 20 min at 3500 rpm using a Hettich UNIVERSAL 320 R centrifuge (Germany). The obtained supernatant was dried in a UN55 Memmert drying oven (Germany) at 105^°^C ± 5^°^C until a constant weight (*S*_2_) was achieved. The WSI (%) was calculated using the following equation:

Water solubility index %=S2−SS1×100.

### 2.6. Determination of Water Activity (*a*_w_)

The *a*_w_ of the samples was measured at 25°C using a *a*_w_ analyzer (Novasina AG LabMaster‐aw, Switzerland).

### 2.7. Determination of Rehydration Capacity

The rehydration capacity of the samples was determined following the method described by [16]. Specifically, 2 g of the sample (*M*_1_) was mixed with 150 mL of pure water and boiled for 10 min in a sealed container. After cooling, the sample was filtered and reweighed (*M*_2_), and the rehydration capacity was calculated using the following equation:

Rehydration capacity=M2M1.

### 2.8. Determination of the Total Phenolic Content

The total phenolic content of the methanolic extracts was determined using a modified Folin–Ciocalteu method [15]. Briefly, 0.4 mL of the extract was mixed with 1.6 mL of a 1:10 (v/v) diluted Folin–Ciocalteu reagent (Sigma‐Aldrich, Germany) in a 15‐mL Falcon tube. The mixture was vortexed (Velp Scientifica F202 A0173, Italy) and left to stand for 5 min. Then, 1.6 mL of a 20% (w/v) sodium carbonate (Na_2_CO_3_) solution was added. After vortexing, the reaction mixture was incubated in the dark for 90 min. The absorbance of the resulting blue complex was measured at 765 nm using a spectrophotometer (Thermo Scientific Evolution 201, United States). A calibration curve was constructed with gallic acid (GA) standards (0–50 mg/L). The total phenolic content was calculated based on the calibration equation *y* = 0.01122*x* + 0.00924 (*R* ^2^ = 0.99801) and expressed as milligrams of GA equivalents GA per 100 g of dry weight.

### 2.9. Determination of Antioxidant Capacity

The antioxidant capacity of the samples was evaluated using the DPPH radical scavenging assay, as described by [15] with slight modifications. Briefly, 0.2 mL of the methanolic extract was added to 3.8 mL of a 0.1 mM DPPH solution (Aldrich, Germany) in ethanol. The mixture was vortexed (Velp Scientifica F202 A0173, Italy) and incubated in the dark for 30 min. The absorbance was then measured at 515 nm against a blank (prepared by replacing the extract with methanol) using a spectrophotometer (Thermo Scientific Evolution 201, United States). The antioxidant capacity was quantified against a Trolox calibration curve (0.1–1.0 mM) with the equation *y* = 0.7392*x* − 1.3707 (*R* ^2^ = 0.9976) and expressed as micromole (*μ*mol) Trolox equivalents per gram of dry weight.

### 2.10. Color Determination

The color of the mango powder samples was analyzed using a Konica Minolta colorimeter (CR‐400, Japan) based on the CIE (International Commission on Illumination) *L*∗*a*∗*b*∗ color system. The *L*∗ axis represents lightness (*L*∗ = 0 for black and *L*∗ = 100 for white), the *a*∗ axis represents the green–red scale (negative values for green, positive for red), and the *b*∗ axis represents the blue–yellow scale (negative values for blue, positive for yellow). As *L*∗, *a*∗, and *b*∗ values do not directly correspond to human visual perception, the chroma (*C*) and hue angle (*α*) were also calculated. The total color difference (*Δ* *E*) was determined to quantify the overall color change during drying. The equations for *C*, *α*, and *Δ* *E* are provided below [17]:

C=a2+b2 ,α=tan−1ba,ΔE=L∗−Lο∗222+a∗−aο∗+b∗−bο∗.

### 2.11. Scanning Electron Microscopy (SEM) Image Acquisition

The effects of different drying applications on the structural properties of powdered mango samples were examined using SEM. SEM images were captured using a Carl Zeiss Gemini 300 (Germany) SEM at the Central Research Laboratory of Bursa Technical University.

### 2.12. Statistical Analysis

In the statistical evaluation of the obtained data, the analysis of variance was performed using the JMP Statistical Discovery Software 7.0 package program (SAS Institute Inc., Cary, United States) and by applying the least significant difference (LSD) multiple comparison test at the confidence interval *α* = 0.95. For total phenolic content and antioxidant capacity, a two‐way factorial ANOVA with factors pretreatment (non‐ULS, ULS15, and ULS30) and drying (200 W, 70°C, and 200 W + 70^°^C), including their interaction, was applied. Data are reported as mean ± SD. Normality and homogeneity were assessed with the Shapiro–Wilk and Levene tests, respectively. Effect sizes are expressed as partial eta squared (*η*p^2^). Statistical significance was set at *α* = 0.05.

## 3. Results and Discussion

### 3.1. Drying Kinetics

Figures 1a, 1b, and 1c show that mango samples dried at 200 W MW without pretreatment had the longest drying time (290 min to reduce moisture from 5.31 to 0.1 g/g dry matter). In contrast, ULS pretreatment (30 min) followed by MW‐HAD at 200 W + 70^°^C achieved the shortest drying time, reducing it by up to 80%. Similarly, [18] reported an 11% reduction in banana drying time with ULS. [19] confirmed the effectiveness of combining ULS and MW. [3, 20, 21] also observed drying time reductions of 12.6% (seaweed), 29.8% (parsley), and 40% (apples), respectively.

Figure 1Moisture content changes over time in mango powder samples dried using different methods: (a) untreated, (b) pretreated with ULS for 15 min, and (c) pretreated with ULS for 30 min.

**Figure:**
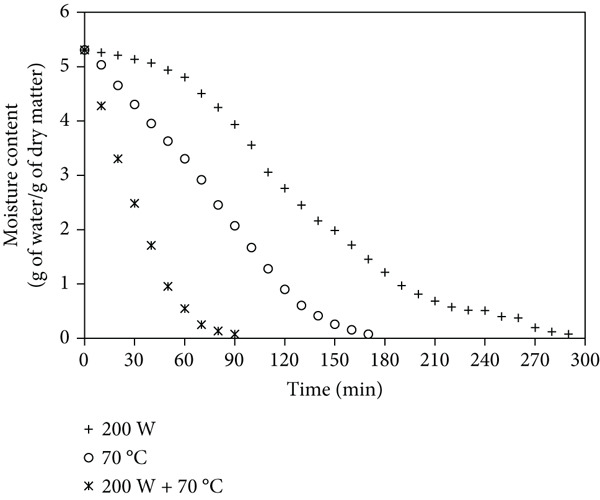
(a)

**Figure:**
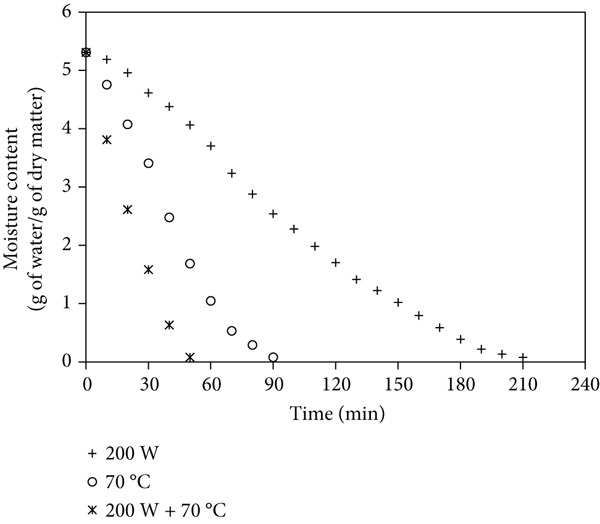
(b)

**Figure:**
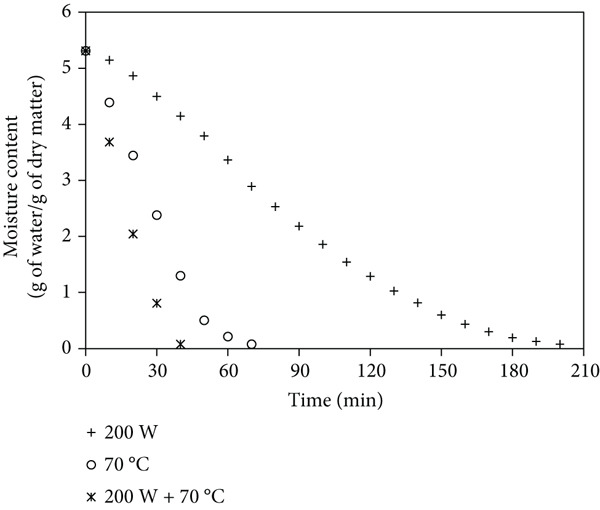
(c)

Figures 2a, 2b, and 2c show the drying rates of mango powder under various drying methods, with and without ULS pretreatment. In MW drying, the rate started relatively low and gradually increased, reaching a distinct peak at intermediate moisture contents. HAD exhibited a moderate initial rate that slightly rose before gradually declining as moisture decreased. In contrast, the combined MW‐HAD method produced a very high initial drying rate that remained elevated during the early‐to‐mid drying stage, likely due to the synergistic effect of rapid internal heating by microwave and continuous surface moisture removal by hot air. This behavior explains the atypical trend observed in the MW‐HAD method compared to the single‐method treatments. ULS pretreatment further enhanced drying rates across all methods by increasing both the initial and peak values, thereby facilitating faster moisture transport and reducing total drying time. [9] reported a nearly fivefold increase in the drying rate of blackberry using ULS (20 min) and HAD. [22] similarly found that ULS (41 W/L, 25 kHz) for 30–60 min improved carrot drying rates at 60°C.

Figure 2Drying rate changes with moisture content in mango powder samples dried using different methods: (a) untreated, (b) pretreated with ULS for 15 min, and (c) pretreated with ULS for 30 min.

**Figure:**
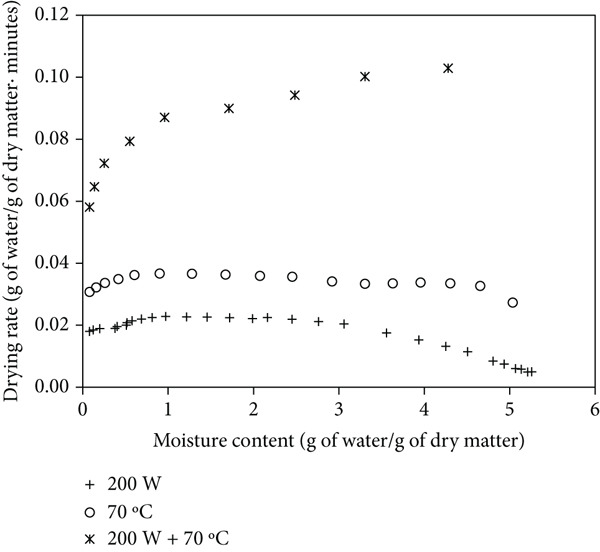
(a)

**Figure:**
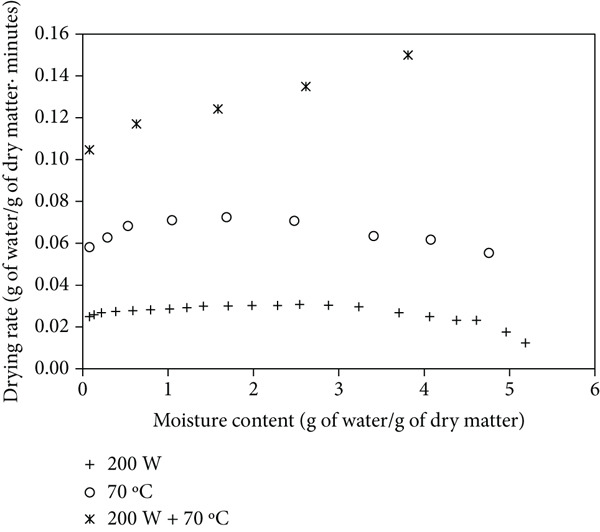
(b)

**Figure:**
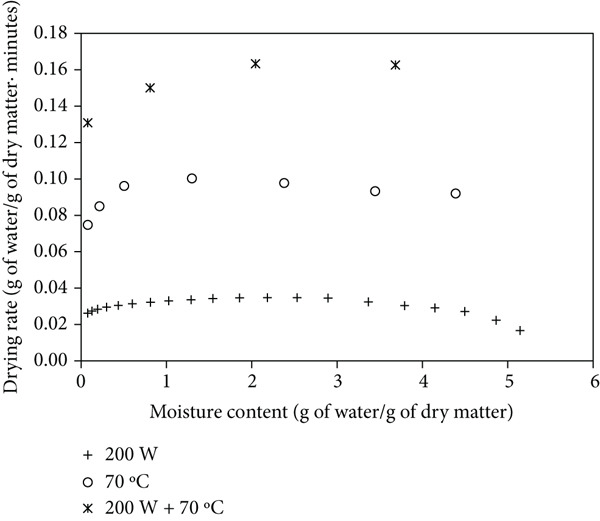
(c)

### 3.2. Physical and Powder Properties

Table 1 shows significant differences (*p* < 0.05) in bulk density, tapped density, and porosity of mango powders according to the drying method. The highest bulk density (0.35 g/cm^3^) was recorded in samples dried at 70°C without ULS pretreatment, whereas the lowest (0.25 g/cm^3^) was found in samples pretreated with ULS for 30 min and dried at 200 W + 70^°^C. Higher bulk density reduces transportation and storage costs, whereas lower density increases oxidation risk, affecting stability. Similar trends were reported by [10] for ULS‐pretreated garlic. [3] also found lower densities and higher porosities in ULS‐pretreated apples.

**Table 1:** Bulk density, tapped density, particle density, and porosity of mango powder samples produced using different drying methods with or without ULS pretreatment.

**ULS pretreatment time**	**Drying parameters**	**Bulk density (g/cm** ^**3**^ **)**	**Tapped density (g/cm** ^**3**^ **)**	**Particle density (g/cm** ^**3**^ **)**	**Porosity**
—	200 W	0.32 ± 0.01^b^	0.56 ± 0.01^bc^	1.36 ± 0.01^c^	58.85 ± 0.25^c^
	70°C	0.35 ± 0.01^a^	0.53 ± 0.02^c^	1.13 ± 0.02^d^	53.11 ± 0.94^e^
	200 W + 70^°^C	0.29 ± 0.01^bcd^	0.59 ± 0.01^abc^	1.36 ± 0.01^c^	56.82 ± 0.36^d^

15 min	200 W	0.30 ± 0.01^bc^	0.59 ± 0.02^ab^	1.59 ± 0.09^ab^	62.85 ± 0.98^ab^
	70°C	0.32 ± 0.01^b^	0.56 ± 0.02^abc^	1.32 ± 0.02^c^	57.12 ± 0.84^cd^
	200 W + 70^°^C	0.27 ± 0.01^de^	0.61 ± 0.01^ab^	1.56 ± 0.01^b^	60.96 ± 0.51^b^

30 min	200 W	0.27 ± 0.02^cde^	0.61 ± 0.01^a^	1.72 ± 0.05^a^	64.27 ± 0.27^a^
	70°C	0.29 ± 0.01^bcd^	0.59 ± 0.02^ab^	1.56 ± 0.04^b^	61.86 ± 0.40^b^
	200 W + 70^°^C	0.25 ± 0.01^e^	0.62 ± 0.03^a^	1.66 ± 0.09^ab^	62.63 ± 0.20^ab^

*Note:* Values with different letters (a–e) within the same column are significantly different at *p* < 0.05.

The tapped density remained largely unchanged in samples pretreated with ULS for 15 min but significantly increased after 30 min of ULS pretreatment followed by MW‐HAD drying. The highest tapped density (0.62 g/cm^3^) was achieved in samples dried at 200 W + 70^°^C, whereas the highest particle density (1.72 g/cm^3^) was observed at 200 W under MW drying after 30 min of pretreatment.

The porosity ranged from 53.11% to 64.27%. The lowest (53.11%) values were observed in samples dried at 70°C without ULS pretreatment, whereas ULS‐pretreated samples (30 min) dried by MW, HAD, and MW‐HAD demonstrated significantly higher values. The highest porosity (64.27%) was observed in samples treated with ULS for 30 min and dried at 200 W.

The flowability (CI) of food powders depends on their particle properties, interparticle forces, bulk density, and moisture content [23]. A positive correlation was observed between CI and cohesiveness (HR) in mango powder (Table 2). Flowability was significantly different (*p* < 0.05) across ULS‐pretreated and nonpretreated samples. The 30‐min ULS pretreatment led to a marked deterioration in flowability, with MW‐HAD‐dried samples exhibiting the highest (CI = 60.01) and (HR = 2.50), indicative of very poor flow characteristics. The mango powders in this study demonstrated higher CI values than Arabian gum (28.42) [24], taro flour (13.96) [10], and pumpkin powder (35.10 ± 0.87) [15]. Similarly, HR values exceeded those reported by [25] for whole milk (1.29), rice oatmeal (1.12), granulated sugar (1.36), and berry powders (1.15). HAD drying without ULS pretreatment demonstrated the lowest CI (33.35 ± 4.19) and HR (1.51 ± 0.09) (Table 2). According to CI and HR classifications [26], all mango powder samples demonstrated medium to very poor flowability (CI > 20) and high cohesiveness (HR > 1.4) across all drying conditions (Table 2).

**Table 2:** Flowability (CI) and cohesiveness (HR) values of mango powder produced using different drying methods with or without ULS pretreatment.

**ULS pretreatment time**	**Drying parameters**	**Flowability (CI) (%)**	**Cohesiveness (HR)**
—	200 W	43.01 ± 0.51^d^	1.75 ± 0.02^cd^
	70°C	33.35 ± 4.19^e^	1.51 ± 0.09^d^
	200 W + 70^°^C	49.67 ± 0.33^bcd^	1.99 ± 0.01^bc^

15 min	200 W	48.66 ± 2.98^bcd^	1.95 ± 0.11^c^
	70°C	43.41 ± 0.74^cd^	1.77 ± 0.02^cd^
	200 W + 70^°^C	56.20 ± 1.44^ab^	2.29 ± 0.08^ab^

30 min	200 W	55.72 ± 4.50^ab^	2.28 ± 0.23^ab^
	70°C	51.01 ± 0.34^bc^	2.04 ± 0.01^bc^
	200 W + 70^°^C	60.01 ± 0.97^a^	2.50 ± 0.06^a^

*Note:* Values with different letters (a–e) within the same column are significantly different at *p* < 0.05.

Samples pretreated with ULS for 30 min and dried at 70°C exhibited the highest WSI of 42.32% (Table 3). Drying influences WSI by converting sugars from crystalline to amorphous forms [27]. Regardless of the drying method or ULS pretreatment, samples dried at 200 W exhibited the highest water holding capacity (WHC) values (Table 3). Mango powders dried at 70°C without ULS pretreatment had the lowest WHC (13.71 g/g), whereas those dried at 200 W after 30 min of ULS pretreatment exhibited the highest WHC (24.47 g/g). WHC is influenced by surface properties, bulk density, and fiber hydrophobicity [28]. Similarly, [15] reported the lowest WHC (4.32 g/g) in pumpkin powders dried by MW‐convective drying at 200 W and 80°C, while the highest WHC (7.57 g/g) was found in powders obtained by convective drying at 60°C.

**Table 3:** Swelling, water holding capacity, and water solubility index of mango powder produced using different drying methods with or without ULS pretreatment.

**ULS pretreatment time**	**Drying parameters**	**Swelling capacity (mL/g)**	**Water holding capacity (g/g)**	**Water solubility index (%)**
—	200 W	19.08 ± 0.33^g^	16.71 ± 0.24^e^	31.27 ± 0.22^g^
	70°C	16.62 ± 0.55^h^	13.71 ± 0.25^h^	33.53 ± 0.09^e^
	200 W + 70^°^C	16.32 ± 0.21^h^	15.50 ± 0.44^fg^	32.57 ± 0.12^f^

15 min	200 W	30.49 ± 0.58^d^	22.45 ± 0.41^b^	33.79 ± 0.17^e^
	70°C	21.77 ± 0.25^f^	15.24 ± 0.23^g^	35.43 ± 0.31^d^
	200 W + 70^°^C	25.49 ± 0.31^e^	18.79 ± 0.53^d^	34.29 ± 0.27^e^

30 min	200 W	48.91 ± 0.51^a^	24.47 ± 0.29^a^	38.01 ± 0.55^c^
	70°C	34.65 ± 0.02^c^	16.64 ± 0.36^ef^	42.32 ± 0.31^a^
	200 W + 70^°^C	43.84 ± 0.21^b^	20.65 ± 0.50^c^	39.78 ± 0.23^b^

*Note:* Values with different letters (a–h) within the same column are significantly different at *p* < 0.05.

Swelling capacity was defined as the volume (milliliters) occupied by 1 g of powder when swollen under specific conditions [29]. As shown in Table 3, the lowest swelling capacity (16.32 mL/g) was observed in samples dried at 200 W + 70^°^C without ULS pretreatment, whereas the highest (48.91 mL/g) was recorded in samples pretreated with ULS for 30 min and dried at 200 W MW (*p* < 0.05). According to [30], low swelling capacity is linked to numerous crystallites that enhance granular stability and limit swelling. In contrast, starch gelatinization disrupts molecular order, increasing starch–water interactions and swelling.

### 3.3. *a*_w_ and Rehydration Capacity

Mango powder samples pretreated with ULS and dried using different methods demonstrated significantly different *a*_w_ values compared with fresh samples (*p* < 0.05) (Table 4). Fresh mango had an *a*_w_ of 0.951. The lowest *a*_w_ (0.248) was observed in untreated samples dried by HAD at 70°C, while the highest (0.296) was recorded in samples pretreated for 30 min and dried at 200 W + 70^°^C (*p* < 0.05). In line with [3], prolonged ULS pretreatment (30 min) increased the *a*_w_ value. [31] similarly reported a 10%–13% increase in *a*_w_ in ULS‐pretreated, convectively dried apple samples. [32] also found higher *a*_w_ values in ULS‐pretreated quince samples than in nontreated quince samples.

**Table 4:** Water activity and rehydration capacity of mango powder produced using different drying methods with or without ULS pretreatment.

**ULS pretreatment time**	**Drying parameters**	**Water activity**	**Rehydration**
	***Fresh***	0.951 ± 0.001^a^	

—	200 W	0.261 ± 0.003^e^	5.16 ± 0.08^b^
	70°C	0.248 ± 0.001^f^	3.67 ± 0.12^f^
	200 W + 70^°^C	0.267 ± 0.006^e^	4.17 ± 0.04^de^

15 min	200 W	0.268 ± 0.003^e^	5.78 ± 0.03^a^
	70°C	0.252 ± 0.001^f^	3.91 ± 0.04^ef^
	200 W + 70^°^C	0.278 ± 0.003^d^	4.57 ± 0.09^c^

30 min	200 W	0.287 ± 0.002^c^	5.26 ± 0.16^b^
	70°C	0.277 ± 0.002^d^	4.08 ± 0.07^de^
	200 W + 70^°^C	0.296 ± 0.001^b^	4.21 ± 0.03^d^

*Note:* Values with different letters (a–f) within the same column are significantly different at *p* < 0.05.

Table 4 shows that mango powder samples pretreated with ULS and dried at 200 W exhibited the highest rehydration capacity (*p* < 0.05), with the maximum value (5.78 ± 0.03) recorded in samples treated for 15 min. This indicates that ULS pretreatment enhances the ability of dried mango powders to regain a near‐fresh state. The rehydration rate primarily depends on cell and structural damage [33], and a higher capacity improves the quality of dried food. Similar trends were reported by [22] for carrots, [34] for mushrooms and okra, and [35] for carrots. In contrast, [3] observed reduced rehydration in ULS‐pretreated apples.

### 3.4. Total Phenolic Content and Antioxidant Capacity

Samples dried at 200 W without ULS pretreatment had the highest total phenolic content (1431.86 mg/100 g GA dry weight) (Table 5). Nevertheless, prolonged ULS pretreatment reduced phenolic compounds. Samples dried at 200 W without ULS pretreatment had higher phenolic content than the fresh samples, although the difference was not statistically significant (*p* > 0.05). In contrast, ULS pretreatment for 30 min combined with HAD resulted in the lowest phenolic content (330.09 mg/100 g GA dry weight) (*p* < 0.05). This aligns with findings in apple slices, where microwave drying preserved or enhanced bioactive compounds, while ULS pretreatment showed no clear benefit and sometimes caused degradation, an effect attributed to prolonged sonication in an aqueous medium [36]. [37] similarly reported a 27% polyphenol loss in ULS‐treated apples. Conversely, [11] found increased phenolic content in dried bananas with higher ultrasonic power. [34, 38] also observed phenolic enhancement in ULS‐pretreated onions and okra.

**Table 5:** Total phenolic content and antioxidant capacity of mango powder produced using different drying methods with or without ULS pretreatment.

**ULS pretreatment time**	**Drying parameters**	**Total phenolic substance (mg/100 g GA dry weight)**	**Antioxidant capacity (*μ*mol Trolox/g dry weight)**
	***Fresh***	1409.09 ± 72.12^a^	30.72 ± 2.23^a^

—	200 W	1431.86 ± 20.86^a^	23.41 ± 0.44^b^
	70°C	502.93 ± 16.75^f^	14.87 ± 0.02^def^
	200 W + 70^°^C	1191.74 ± 31.58^b^	18.51 ± 0.18^c^

15 min	200 W	1021.54 ± 17.14^c^	18.65 ± 0.17^c^
	70°C	360.46 ± 6.04^g^	14.29 ± 0.19^ef^
	200 W + 70^°^C	889.63 ± 8.25^d^	17.64 ± 0.10^c^

30 min	200 W	919.91 ± 8.10^d^	16.41 ± 0.12^cde^
	70°C	330.09 ± 7.83^g^	13.46 ± 0.09^f^
	200 W + 70^°^C	748.19 ± 9.74^e^	16.83 ± 0.01^cd^

*Note:* Values with different letters (a–g) within the same column are significantly different at *p* < 0.05.

The antioxidant capacity of mango powder decreased as the duration of ULS pretreatment increased (Table 5). Total phenolic content and antioxidant capacity are directly correlated with fruit and vegetable consumption [39]. The highest antioxidant capacity (23.41 *μ*mol Trolox/g dry weight) was recorded in samples dried at 200 W without ULS pretreatment, although it remained significantly lower than that of the fresh sample (30.72 ± 2.23 *μ*mol Trolox/g dry weight) (*p* < 0.05). Similar trends were reported by [38], who found that ULS pretreatment (5 min) reduced antioxidant activity, and by [40]. Nevertheless, [34] reported that combining ULS with vacuum treatment slightly increased antioxidant activity compared to controls. From a technological perspective, ULS pretreatment offers benefits, but significant losses of bioactive compounds have been reported, especially when sonication is applied in liquid media [41].

A two‐way factorial ANOVA (pretreatment: non‐ULS, ULS15, and ULS30; drying: 200 W, 70°C, and 200 W + 70^°^C) showed that, for total phenolic content, the drying method accounted for the largest share of variance (*F*(2, 9) = 1655.46, *p* < 0.0001, partial *η* ^2^ = 0.997), followed by pretreatment (*F*(2, 9) = 445.53, *p* < 0.0001, partial *η* ^2^ = 0.990). The pretreatment × drying interaction was significant (*F*(4, 9) = 34.39, *p* < 0.0001, partial *η* ^2^ = 0.939), indicating method‐dependent gains from ULS. For antioxidant capacity, the drying method again had the greater effect (*F*(2, 9) = 608.18, *p* < 0.0001, partial *η* ^2^ = 0.993) relative to pretreatment (*F*(2, 9) = 243.16, *p* < 0.0001, partial *η* ^2^ = 0.982), with a significant interaction (*F*(4, 9) = 75.49, *p* < 0.0001, partial *η* ^2^ = 0.971). Residuals satisfied normality (Shapiro–Wilk *p* > 0.83); Levene′s test indicated heteroscedasticity across cells, but the balanced design renders the F‐tests robust. Overall, the drying method exerted the dominant influence on both outcomes, with a significant pretreatment × drying interaction, indicating that the benefit of ULS pretreatment depends on the drying method.

### 3.5. Color Values

Color is a key quality attribute of dried fruits, as browning indicates spoilage from enzymatic or nonenzymatic reactions [42]. In this study, fresh mango had an *L*∗ value of 80.01, whereas dried powders ranged from 53.88 to 70.50 (Table 6). The dried mango samples had lower brightness than the fresh mango. The highest *L*∗ value was observed in samples dried at 200 W + 70^°^C with a 30‐min ULS pretreatment (*p* < 0.05), indicating better brightness. Similar findings were reported by [31, 38]. Additionally, [43] reported that increasing ULS pretreatment further enhanced the *L*∗ and *a*∗ values in hot air–microwave–dried tomatoes.

**Table 6:** Color parameters of mango powder produced using different drying methods with or without ULS pretreatment.

**ULS pretreatment time**	**Drying parameters**	**L**∗	**a**∗	**b**∗	**C**	**α** ^°^	**Δ** **E**
	***Fresh***	80.01 ± 0.59^a^	4.75 ± 0.56^h^	62.72 ± 0.29^a^	62.90 ± 0.30^a^	85.72 ± 0.51^a^	

—	200 W	53.88 ± 1.32^h^	13.46 ± 0.41^a^	41.75 ± 1.10^i^	43.87 ± 1.01^h^	72.15 ± 0.79^h^	31.10 ± 1.16^a^
	70°C	62.97 ± 0.99^f^	10.92 ± 0.25^c^	53.55 ± 1.01^f^	54.65 ± 0.98^e^	78.51 ± 0.38^f^	16.39 ± 1.01^cd^
	200 W + 70^°^C	65.91 ± 0.36^d^	8.10 ± 0.46^d^	48.85 ± 0.40^g^	49.52 ± 0.41^f^	80.62 ± 0.53^e^	17.01 ± 0.35^c^

15 min	200 W	57.12 ± 0.77^g^	12.14 ± 0.52^b^	44.72 ± 0.43^h^	46.35 ± 0.48^g^	74.85 ± 0.57^g^	26.51 ± 0.32^b^
	70°C	64.22 ± 0.91^e^	8.58 ± 0.47^d^	55.77 ± 0.63^d^	56.43 ± 0.63^d^	81.30 ± 0.48^d^	13.53 ± 0.98^e^
	200 W + 70^°^C	68.61 ± 0.60^c^	6.69 ± 0.28^g^	54.64 ± 0.44^e^	55.05 ± 0.43^e^	83.06 ± 0.31^c^	10.64 ± 0.46^f^

30 min	200 W	62.10 ± 0.68^f^	8.53 ± 0.29^d^	54.69 ± 0.74^e^	55.35 ± 0.73^e^	81.18 ± 0.33^de^	15.84 ± 0.67^d^
	70°C	67.60 ± 0.61^c^	7.34 ± 0.08^e^	59.39 ± 0.69^b^	59.84 ± 0.68^b^	82.99 ± 0.12^c^	8.71 ± 0.64^g^
	200 W + 70^°^C	70.50 ± 0.75^b^	5.58 ± 0.31^g^	58.37 ± 0.53^c^	58.64 ± 0.55^c^	84.58 ± 0.26^b^	6.61 ± 0.54^h^

*Note:* Values with different letters (a–i) within the same column are significantly different at *p* < 0.05.

Mango powders dried at 70°C with a 30‐min ULS pretreatment also demonstrated *b*∗ and chroma (*C*∗) values closest to those of fresh mango. In contrast, samples dried at 200 W without ULS pretreatment had the lowest *L*∗ and *C*∗ values. Additionally, ULS‐treated samples at 200 W + 70^°^C for 30 min exhibited the lowest *Δ* *E* value, reflecting minimal color change (*p* < 0.05).

Dried mango powders in this study showed lower overall color attributes compared to fresh mango. The color changes may result from carotenoid degradation, oxidation, and browning reactions [44]. Nevertheless, ULS pretreatment minimized further color loss in this study, with longer pretreatment levels resulting in higher *L*∗, *b*∗, and chroma (*C*∗) values and lower *a*∗ values, closer to those of fresh mango (Table 6). This effect can be explained by ULS‐induced microstructural changes, which create microchannels that enhance mass transfer and reduce drying time [45]. Shorter drying time limits the time the samples are exposed to heat and oxygen, thereby reducing nonenzymatic browning and carotenoid degradation. The overall color difference (*Δ* *E*) was lower in ULS‐pretreated samples, indicating that this treatment maintained visual quality more effectively by preserving lightness and yellowness while minimizing undesirable browning reactions. [46] reported that ultrasonically pretreated eggplant slices retained better surface color and exhibited a lower *Δ* *E*.

### 3.6. SEM Analysis

SEM images (Figures 3a, 3b, and 3c) demonstrated distinct structural differences based on the drying method and ULS pretreatment time. Samples dried at 70°C without pretreatment had a homogeneous structure with small pores. In contrast, ULS‐pretreated samples, especially those dried using HAD, exhibited larger pores and visible structural damage. The SEM images revealed that 15‐min ULS pretreatment produced more porous structures, whereas 30‐min treatment caused significant structural destruction due to ULS cavitation. A similar pattern was noted by [3] for dried apples.

Figure 3SEM images of mango samples dried using different methods: (a) untreated, (b) pretreated with ULS for 15 min, and (c) pretreated with ULS for 30 min.

**Figure:**
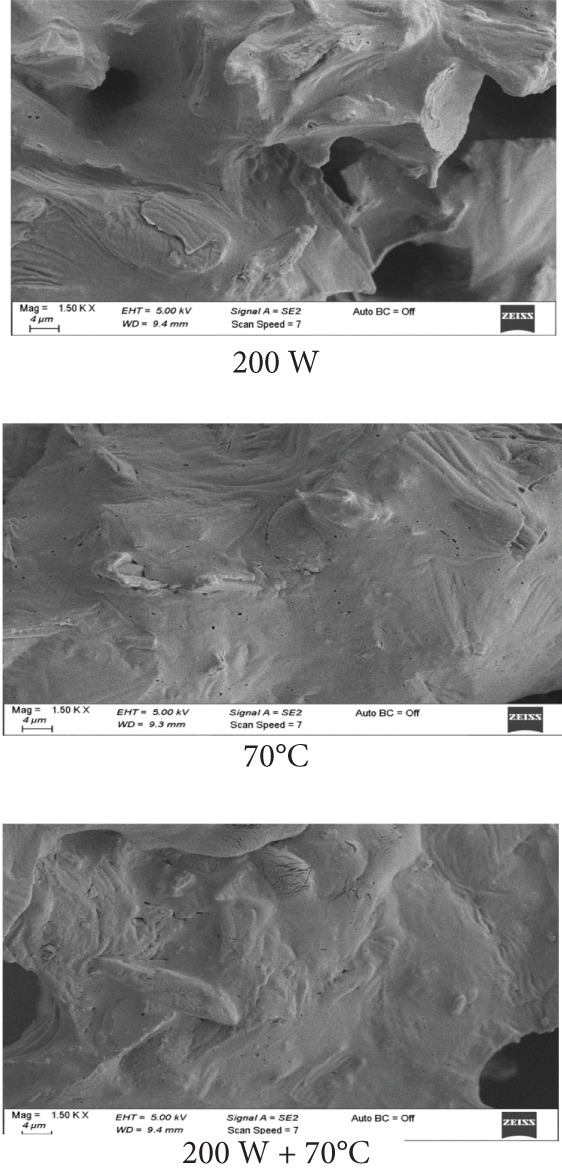
(a)

**Figure:**
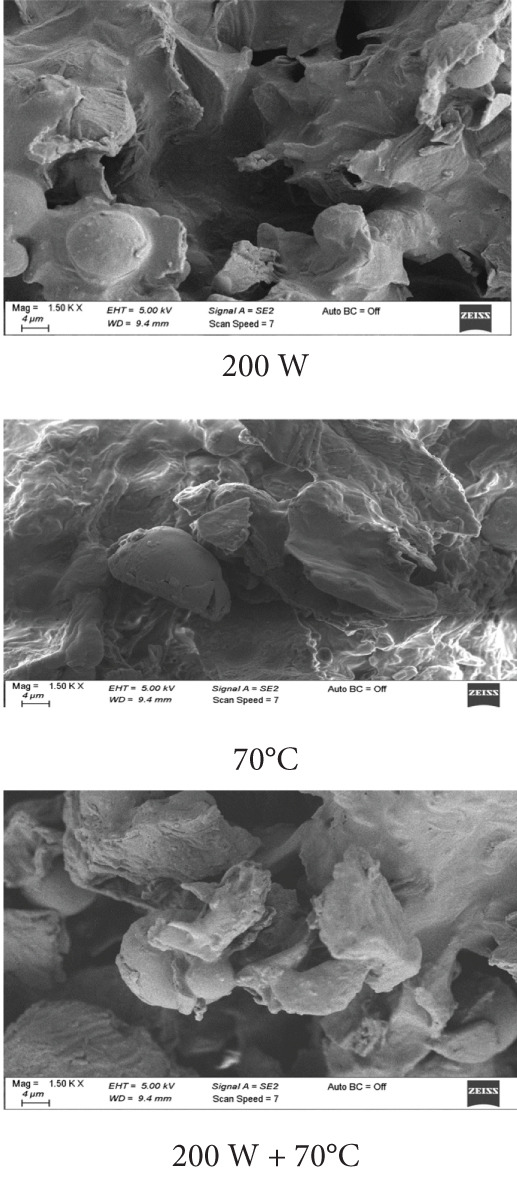
(b)

**Figure:**
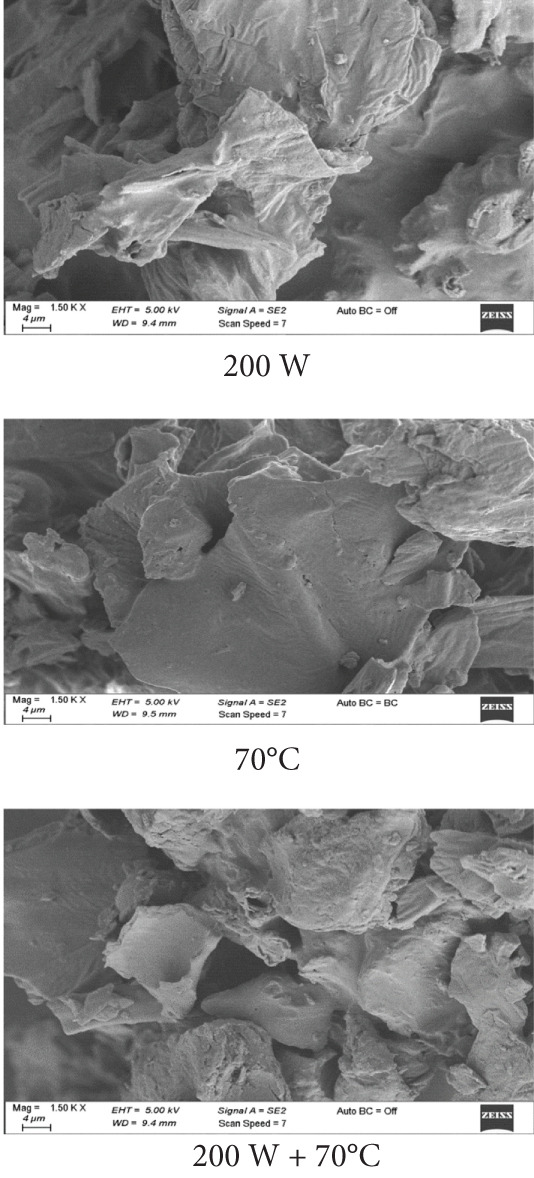
(c)

Prolonged ULS increased the porosity by creating microchannels, which affected the powder density and physical properties. Previous studies have confirmed that ULS forms microscopic channels in plant tissues [47–49]. [50] reported that higher ULS power produced larger intercellular gaps, porosity, and surface tears in dried orange peels. These channels improve water movement, thus accelerating drying [51].

## 4. Conclusion

This study reveals that drying method selection for mango powder requires careful consideration of targeted quality parameters. While ULS pretreatment (30 min) with MW‐HAD (200 W + 70^°^C) offers unparalleled drying efficiency (80% time reduction), MW drying at 200 W without pretreatment emerges as the optimal approach for nutrient preservation, maintaining phenolic and antioxidant levels statistically equivalent to fresh mango (*p* > 0.05). Conventional HAD remains preferable when superior bulk density (0.35 g/cm^3^) and (CI = 33.35) are critical for powder handling and storage. Future research should optimize ULS parameters to mitigate quality degradation while maintaining efficiency benefits.

## Disclosure

This publication is based on the master′s thesis of Ahmed Abdirahman Ahmed submitted to Bursa Technical University [52].

## Conflicts of Interest

The authors declare no conflicts of interest

## Funding

No funding was received for this manuscript.

## Data Availability

The data that support the findings of this study are available from the corresponding author upon reasonable request.
